# Perceived Impact of Covid-19 Across Different Mental Disorders: A Study on Disorder-Specific Symptoms, Psychosocial Stress and Behavior

**DOI:** 10.3389/fpsyg.2020.586246

**Published:** 2020-11-17

**Authors:** Hannah L. Quittkat, Rainer Düsing, Friederike-Johanna Holtmann, Ulrike Buhlmann, Jennifer Svaldi, Silja Vocks

**Affiliations:** ^1^Department of Clinical Psychology and Psychotherapy, Osnabrück University, Osnabrück, Germany; ^2^Department of Research Methodology, Diagnostics and Evaluation, Osnabrück University, Osnabrück, Germany; ^3^Department of Clinical Psychology and Psychotherapy, University of Münster, Münster, Germany; ^4^Department of Clinical Psychology and Psychotherapy, University of Tübingen, Tübingen, Germany

**Keywords:** corona, Covid-19, mental disorders, impact, stress, symptom severity, pandemics

## Abstract

The recent outbreak of the coronavirus disease (Covid-19) has plunged countries across the world into crisis. Both in the general population and in specific subgroups such as infected people or health care workers, studies have reported increased symptoms of anxiety, depression and stress. However, the reactions of individuals with mental disorders to Covid-19 have largely been neglected. The present study therefore aimed to investigate the perceived impact of Covid-19 and its psychological consequences on people with mental disorders. In this online survey, participants were asked to evaluate their disorder-specific symptoms, perceived psychosocial stress and behaviors related to Covid-19 in the current situation and retrospectively before the spread of Covid-19. The study included participants with self-identified generalized anxiety disorder (GAD), panic disorder and agoraphobia (PA), illness anxiety disorder (IA), social anxiety disorder (SAD), depression (DP), obsessive–compulsive disorder (OCD), body dysmorphic disorder (BDD), eating disorders (ED), schizophrenia spectrum and other psychotic disorders (SP), other non-specified mental disorder (other) as well as mentally healthy controls (HC). The results of bayesian parameter estimation suggest that the symptom severity of DP, GAD, IA and BDD has deteriorated as a reaction to Covid-19. Across all mental disorders and HC, self-reported psychosocial stress levels were higher during the outbreak of Covid-19 compared to before. A reduced frequency of social contacts and grocery shopping was found for all participants. People with self-identified mental disorders showed higher personal worries about Covid-19 and a higher fear of contagion with Covid-19 than did HC. According to our findings, Covid-19 may reinforce symptom severity and psychosocial stress in individuals with mental disorders. In times of pandemics, special support is needed to assist people with mental disorders and to prevent symptom deterioration.

## Introduction

More than a third of the total population of the European Union is affected by a mental disorder (Wittchen et al., 2011). With the recent pandemic of coronavirus disease (Covid-19), people with and without mental disorders are facing profound changes to their lives, such as quarantine and isolation (e.g., Kaparounaki et al., 2020; Li et al., 2020a; Zhu et al., 2020). Yet, it is unknown how Covid-19 is impacting the psychological health of people with mental disorders.

In the past, research has mostly examined the perceived impact of epidemics and pandemics on mental health in the general population (e.g., Lau et al., 2005; Taylor et al., 2008) or in subgroups such as infected persons (e.g., Chua et al., 2004; Lee et al., 2007), people undergoing quarantine or isolation (e.g., Hawryluck et al., 2004; Wang et al., 2011; Brooks et al., 2020) or health care workers (e.g., Tam et al., 2004; Ko et al., 2006; Liu et al., 2012; Kisely et al., 2020). For example, it was found that Hong Kong residents who felt helpless, apprehensive and horrified during the outbreak of the severe acute respiratory syndrome related to coronavirus (SARS) in 2003 were more likely to report posttraumatic stress symptoms than those who did not have these feelings (Lau et al., 2005). Another study on the SARS epidemic in Hong Kong revealed that participants with a higher perceived likelihood of contracting a SARS infection reported significantly greater anxiety scores compared to those with a lower perceived likelihood (Leung et al., 2005). Furthermore, a study on equine influenza in Australia found that people living in areas with a high risk of infection had a greater risk of high psychological distress than those living in uninfected areas (Taylor et al., 2008). Hence, people living in an infected area may feel negatively affected by the outbreak of an epidemic.

During epidemics, certain subgroups, such as survivors, quarantined people or health care workers, seem to show similar results regarding the psychological consequences. As such, one investigation on the survivors of SARS showed higher stress level symptoms in these survivors during the outbreak compared to a matched healthy control group (Lee et al., 2007). Moreover, these symptoms persisted over a 1-year follow-up, especially in health care workers who were SARS survivors (Lee et al., 2007). Other studies on the effects of being quarantined and isolated have also reported high levels of stress symptoms and exhaustion among quarantined health care workers (Bai et al., 2004) and depressive symptoms among quarantined persons (Hawryluck et al., 2004), even up to 3 years after being quarantined (Liu et al., 2012). A recent review (Brooks et al., 2020) on the psychological impact of quarantine and isolation described negative psychological effects in terms of anger, posttraumatic stress symptoms, insomnia, avoidance behaviors and confusion, highlighting the importance of reducing quarantine to a minimally required period of time.

Despite the described negative emotional consequences of epidemics in the general population, studies in individuals with mental disorders are surprisingly rare. In former SARS patients in Hong Kong, a cumulative incidence of 58.9% for any DSM-IV mental disorder was found 30 months after the SARS outbreak, as well as a fairly high prevalence (33.33%) of mental disorders (Mak et al., 2009). In addition, all SARS survivors reported a lower health-related quality of life compared to established norms for the general population (Mak et al., 2009). Another study (Jeong et al., 2016) reported a higher risk of experiencing anger and anxiety in people with a history of mental disorders 4 – 6 months after being isolated due to a possible infection with the Middle East Respiratory Syndrome (MERS). Further, in a study examining different groups, SARS patients stated higher levels of anxiety and depressive symptoms than did a community sample, but lower levels than patients with a depressive or anxiety diagnosis (Cheng et al., 2004). Unfortunately, none of the aforementioned studies on epidemics investigated participants with mental disorders which already existed prior to the outbreak. Moreover, Jeong et al. (2016) did not specify in their study whether or not the participants with a history of mental disorders were still suffering from a mental disorder at the time of the investigation. Thus, it remains unclear how persons with pre-existing mental illness are affected by the outbreak of an epidemic or pandemic.

Recent findings on Covid-19 in the general population seem to underline the results of studies on earlier pandemics, such as the occurrence of anxiety and depressive symptoms as well as high stress levels after the outbreak (e.g., Mazza et al., 2020; Odriozola-González et al., 2020; Wang et al., 2020b). For instance, in a study on the psychological impact of the early-stage Covid-19 pandemic on the general Chinese population, 53.8% of the participants rated the perceived psychological impact of Covid-19 as moderate to severe (Wang et al., 2020a). Another study during the initial outbreak of Covid-19 in the general Chinese population found significantly higher values on all scales of the symptom checklist (SCL-90) compared to previously established norms (Tian et al., 2020), indicating an increase of the perceived psychological burden in the general population. Further studies underline this assumption, reporting other psychological symptoms in the general population associated with the pandemic, such as sleep problems (Li et al., 2020b), increased dependence on internet use (Sun et al., 2020) or worries about financial issues (Tull et al., 2020) and the economy (Betsch et al., 2020). Moreover, studies in persons who had contracted Covid-19 have reached similar results. One study reported an increased prevalence of depressive symptoms in patients with Covid-19 compared to participants living in isolation who had not been infected (Zhang et al., 2020). Other studies examined a positive association between higher levels of anxiety as well as posttraumatic stress and having an infected family member (González-Sanguino et al., 2020; Mazza et al., 2020). In sum, Covid-19 seems to place a psychological burden on the general population similar to that indicated in earlier epidemics and pandemics.

Notably, even though individuals with mental disorders might be particularly vulnerable with respect to the consequences of Covid-19 (Fiorillo and Gorwood, 2020), most of the current studies did not specifically address individuals with mental disorders. However, these individuals face various burdens in their daily lives, such as a reduced life expectancy (Chesney et al., 2014), stigmatization (e.g., Reavley and Jorm, 2011; Serafini et al., 2011) and role impairment (Kessler et al., 2009). These experiences might be enhanced by the outbreak of Covid-19. Therefore, during times of Covid-19, particularly individuals with mental disorders may undergo difficulties in accessing mental health care services, may suffer from reduced social interactions and may experience severe emotional responses to the pandemic, such as increased feelings of loneliness (Fiorillo and Gorwood, 2020; Yao et al., 2020). For instance, a study by Davide et al. (2020) reported higher obsessions and compulsions in patients with obsessive–compulsive disorder (OCD) while being quarantined during the outbreak of Covid-19 relative to before the outbreak. Furthermore, reported contamination symptoms as well as a remitted OCD before the quarantine were associated with increased OCD symptoms during quarantine than before (Davide et al., 2020). Other studies have supported these results, finding symptom deteriorations and/or increased relapse rates in mental disorders such as alcohol substance use (Sun et al., 2020), eating disorders (ED; Castellini et al., 2020), and hospitalized patients with schizophrenia who were suspected to have contracted Covid-19 (Liu et al., 2020). Finally, a recent review suggests the onset of a psychotic episode during Covid-19 to be associated with psychosocial stress (Brown et al., 2020). These very limited studies emphasize the assumed negative impact of the Covid-19 outbreak on mental disorders.

To our knowledge, no study to date has investigated the influence of Covid-19 on disorder-specific symptoms in individuals with different mental disorders. Although a recent study found that previous diagnoses of mental disorders were significantly associated with current depression, anxiety and posttraumatic stress symptoms during the Covid-19 pandemic (González-Sanguino et al., 2020), the authors did not evaluate disorder-specific symptoms currently and before the outbreak. Therefore, the aim of the present study was to investigate the perceived impact of Covid-19 and its psychological consequences on individuals with different mental disorders. We intended to examine persons with generalized anxiety disorder (GAD), panic disorder and agoraphobia (PA), illness anxiety disorder (IA), social anxiety disorder (SAD), depression (DP), OCD, body dysmorphic disorder (BDD), ED, schizophrenia spectrum and other psychotic disorders (SP) as well as healthy controls (HC). Due to the lack of studies on mental disorders in relation to pandemics, we sought to examine, from an exploratory perspective, possible changes in symptom severity, perceived stress levels, and behaviors related to Covid-19 in individuals with mental disorders. Furthermore, to identify possible differences between people with and without mental disorders, we aimed to compare perceived stress levels, the number of corona-related behaviors, worries and fears as well as perceived changes in quality of life between the two groups.

## Materials and Methods

### Recruitment and Participants

For data collection, an online survey was implemented via Unipark (version fall 2019, Questback GmbH, Cologne, Germany). Inclusion criteria were sufficient German-language skills, age of 18 years or older, being mentally healthy, or the experience of one or more of the following mental disorders: GAD, PA, IAD, SAD, DP, OCD, BDD, ED, SP. The sample was recruited through university press releases and e-mail lists, flyer, social media, institutions for education in psychotherapy, outpatient departments, mental hospitals, psychotherapist associations, self-help groups and assisted living departments. Data were collected from April 2 to May 6 2020 during the lockdown in Germany, as during that time period, restrictions on daily life were applied to all citizens, such as travel bans, wearing a mask while grocery shopping, bans on visiting others, keeping a distance of 1.5 m from other people, stay-at-home advice, cancelation of all major events and closing of all restaurants, shops, fitness studios and public swimming pools (German Chancellor, 2020).

A total of *N* = 7933 persons opened the homepage of the survey, of whom *n* = 3101 confirmed their consent for participation. Of these, *n* = 2267 (73.11%) finished the study. From this sample, *n* = 4 participants were excluded due to ambiguous details about their mental health and *n* = 4 participants were excluded because they did not meet the age criterion (<18 age years old). Moreover, *n* = 26 participants were excluded because they participated after May 3 2020. This exclusion was set due to the first easing of restrictions, which were announced on May 4 2020 by several Federal states of Germany. Thus, the final sample consisted of *n* = 2233 persons.

From the final sample, *n* = 830 (37.17%) participants reported that they were suffering from a current mental disorder. Of those who were not currently suffering from a mental disorder, *n* = 377 (26.87%) reported having been affected by a mental disorder in the past. Of those with a mental disorder, *n* = 581 (48.14%) reported that they were in outpatient treatment, while *n* = 17 (1.41%) were in inpatient treatment. Of the total sample, *n* = 975 participants (43.66%) reported a past inpatient or outpatient treatment and *n* = 435 (19.48%) a current pharmacological treatment due to a mental disorder. Of those who reported no current pharmacological treatment, *n* = 289 (16.07%) stated that they had received pharmacological treatment in the past.

Of the final sample, *n* = 135 (6.05%) participants identified themselves as currently suffering from GAD, *n* = 83 (3.72%) from PA, *n* = 30 (1.34%) from IA, *n* = 86 (3.85%) from SAD, *n* = 586 (26.24%) from DP, *n* = 47 (2.11%) from OCD, *n* = 16 (0.72%) from BDD *n* = 62 (2.78%) from ED, and *n* = 6 (0.27%) from SP. If a mental disorder (current or past) was reported but none of the described disorders were selected, participants were labeled as other non-specified mental disorder (other, *n* = 156, 6.99%). A number of *n* = 1026 (45.95%) without any current and past mental disorder identified themselves as HC.

### Procedure

To access the study website, the participants could either scan a QR code or use a web link. The landing page included information about the aim, duration (around 20–30 min), inclusion criteria as well as privacy and confidentiality issues of the study. Once the participants provided informed consent by agreeing to the aforementioned aspects, a questionnaire assessing demographic data and mental health was presented. If participants reported a current or a past mental disorder, they were asked to self-identify the respective mental disorder by reading short descriptions of the disorders, based on the main criteria of the Diagnostic and Statistical Manual of Mental Disorders – fifth edition (American Psychiatric Association, 2018), and stating whether they were suffering from the described disorder at the time of participation. In the case of comorbidity, participants were requested to choose the disorder with the highest burden at the time of participation. Following this, disorder-specific questionnaires were administered to the participants with the respective diagnosis. Additionally, all participants were asked about their perceived stress during the past 4 weeks, followed by a questionnaire on the current situation surrounding Covid-19 (e.g., social contact, hand washing, grocery shopping).

To assess the situation before the spread of Covid-19, participants were instructed to respond to the same questionnaires retrospectively for November 2019. To support participants’ recollection of their thoughts, feelings and behavior, they were asked to recall the number of life events with the help of their calendars, photos on their smartphones, and diaries from November 2019 before answering the questionnaires retrospectively. Healthy participants only answered the questionnaire on perceived stress and the questionnaire on the situation surrounding Covid-19 for the current time and for November 2019 retrospectively. The retrospective evaluation of symptoms for November 2019 was defined as T_0_, whereas the current evaluation of symptoms was defined as T_1_. The study was conducted in accordance with the Declaration of Helsinki and was approved by the local ethics of the University of Münster.

### Measures

#### Body Dysmorphic Symptoms Inventory (German: Fragebogen Körperdysmorpher Symptome, FKS)

The FKS (Buhlmann et al., 2009) is a self-report questionnaire assessing body dysmorphic symptoms in the last week with 18 items rated on a 5-point Likert scale (0 = *not at all/never/don’t even think about it* to 4 = *very strong/more than five times a day/more than 8 h a day*). The internal consistency of this questionnaire was found to be α = 0.88 in the original study (Buhlmann et al., 2009), and α = 0.76 at T_0_ and α = 0.66 at T_1_ in the present study.

#### Continuum of Auditory Hallucinations – State Assessment (CAHSA)

The CAHSA (Schlier et al., 2017) consists of nine items assessing hallucination spectrum experiences, namely vivid imagination, intrusive thoughts, perceptual sensitivity and auditory hallucinations, rated on an 11-point Likert scale (0 = *not at all* to 10 = *very much*). In the present study, items referred to the last 4 weeks. Internal consistency in our sample was α = 0.76 at T_0_ and α = 0.54 at T_1_.

#### Depression Anxiety Stress Scales – Depression Subscale (DASS-D)

The DASS (Lovibond and Lovibond, 1995; German-language version: Nilges and Essau, 2015) assesses depressive mood over the past week using seven items rated on a 4-point Likert scale (0 = *never* to 3 = *always*) with high internal consistency in the original study (α = 0.88; Nilges and Essau, 2015) and the present study (T_0_: α = 0.93, T_1_: α = 0.88).

#### Eating Disorder Examination-Questionnaire – 2nd Edition (EDE-Q)

The EDE-Q (Fairburn et al., 2014; German-language version: Hilbert and Tuschen-Caffier, 2016) assesses the psychopathology of eating disorders during the past 28 days with 22 items belonging to four subscales, namely *eating concern, restraint, shape concern* and *weight concern*, rated on a 7-point Likert scale (0 = *no days*/*never*/*not at all* to 6 = *every day*/*every time*/*markedly*). The internal consistency of this questionnaire was found to be high for the subscales and the total score in the original study (0.85 ≤ α ≤ 0.97; Hilbert et al., 2007). In the present study, the internal consistencies were acceptable to excellent for the total score and for the subscales (total score: T_0_: α = 0.93, T_1_: α = 0.92, restraint: T_0_: α = 0.89, T_1_: α = 0.84; eating concern: T_0_: α = 0.73, T_1_: α = 0.76; shape concern: T_0_: α = 0.76, T_1_: α = 0.77; weight concern: T_0_: α = 0.80, T_1_: α = 0.78).

#### Patient Health Questionnaire – Panic Module and Stress Subscale (PHQ)

The PHQ – Panic and Stress Subscale (Spitzer et al., 1999; German-language version: Löwe et al., 2002) are screening tools, based on diagnostic criteria from the DSM-IV. The panic subscale assesses the diagnostic criteria of a panic disorder and physical symptoms during a panic attack. It consists of 15 items, which are answered dichotomously with *yes* or *no* with good classification properties (sensitivity of 73% and specificity of 98% in medical patients; Gräfe et al., 2004). In this study, if the criteria for experiencing a panic attack in the last 4 weeks were met, symptom severity was measured by calculating the sum of all items (*n* = 11), which examined physical symptoms of a panic attack. The stress subscale consists of ten items (3-point Likert scale; 0 = *not affected* to 2 = *severely affected*), asking about psychosocial stress factors during the last 4 weeks that indicate triggering or maintaining conditions of a mental disorder. Internal consistencies were α = 0.73 at T_0_ and α = 0.69 at T_1_.

#### Penn State Worry Questionnaire (PSWQ-d)

The PSWQ-d (Meyer et al., 1990; German-language version: Glöckner-Rist and Rist, 2014) assesses excessive, unrealistic concern as a central cognitive concomitant syndrome of a GAD using 16 items rated on a 5-point Likert scale (1 = *not at all typical of me* to 5 = *very typical of me*). High internal consistency was found for the German-language version (α = 0.86, Stöber, 1995) as well as for the current study (T_0_: α = 0.89, T_1_: α = 0.85).

#### Questions on the Situation Surrounding Covid-19

To evaluate the current living conditions, thoughts and feelings regarding Covid-19, questions relating to the following topics were presented: Covid-19 infection (current or past), staying at home most of the time due to Covid-19, worries about the consequences of Covid-19 personally and for society (from 1 = *not at all* to 5 = *strongly*), estimated likelihood of becoming infected with SARS-CoV-2, the perceived fear of contracting an infection (from 1 = *very little* to 5 = *very high*), their number of direct social contacts per week, the time spent on obtaining information about Covid-19 in minutes per day, the frequency of hand washing per day, the time spent on hand washing in minutes per day, the frequency of hand disinfection per day, the frequency of grocery shopping per week. The latter four questions were also assessed retrospectively for November 2019. Additionally, all participants were asked about changes regarding their quality of life (1 = *considerably improved* to 5 = *considerably worsened*), while only the participants with a mental disorder were further asked about the perceived changes concerning their mental health (1 = *considerably improved* to 5 = *considerably worsened*) and an increased need for therapeutic support (0 = *no*, 1 = *yes*) due to Covid-19.

#### Socio-Demographic Characteristics

All participants completed a questionnaire collecting demographic data such as gender, age, educational level, relationship status, size and structure of their home, the assessment of mental disorders, current or past outpatient psychotherapy, current or past inpatient psychotherapy and current pharmacological treatment.

#### Social Interaction Anxiety Scale (SIAS) and Social Phobia Scale (SPS)

The SIAS (Mattick and Clarke, 1998; German-language version: Stangier et al., 1999) captures anxiety in situations of social interaction using 20 items rated on a 5-point Likert scale (0 = *not at all* to 4 = *very much*). The SPS (Mattick and Clarke, 1998; German-language version: Stangier et al., 1999) refers to anxiety in situations where an action could be observed by others, such as public speaking, with 20 items rated on the same 5-point Likert scale as the SIAS. Internal consistency was found to be high, both for the SIAS (α = 0.94) and the SPS (α = 0.94) in a sample of patients with SP (Stangier et al., 1999) as well as in the present study (SIAS: T_0_: α = 0.93, T_1_: α = 0.92; SPS: α = 0.94 at T_0_ and α = 0.93 at T_1_).

#### Whitely Index (WI)

The WI (Pilowsky, 1967; German-language version: Glöckner-Rist et al., 2014) consists of 14 items assessing attitudes and beliefs of people with illness anxiety. Items are answered dichotomously (0 = *no* or 1 = *yes*). For the German-language version, internal consistency was α = 0.83 (Hinz et al., 2003). In the present study, internal consistency was *ρ_*KR*_*_20_ = 0.80 at T_0_ and *ρ_*KR*_*_20_ = 0.60 at T_1_.

#### Yale-Brown Obsessive Compulsive Scale – Symptom Checklist (Y-BOCS)

The symptom checklist of the Y-BOCS (Goodman et al., 1989; German-language version: Hand and Büttner-Westphal, 1991) was used as a self-report measure to assess obsessive-compulsive symptoms during the last 7 days. The scale consists of ten items rated on a 5-point Likert scale (0–4) with differing labels (Hand and Büttner-Westphal, 1991). High internal consistency was found for the German-language version (α = 0.80 for the total score, Jacobsen et al., 2003) as well as in our study (total score: T_0_: α = 0.94; T_1_: α = 0.93; obsession subscale: T_0_: α = 0.90; T_1_: α = 0.85; compulsion subscale: T_0_: α = 0.93, T_1_: α = 0.91).

### Data Analysis

Data were analyzed using the software IBM SPSS Statistics (version 26) for descriptive analysis, and for inferential Bayesian statistics the software R (version 3.5.3) and RStudio (version 1.1.463) with the packages rstanarm (version 2.21.1, Goodrich et al., 2020), rjags (version 4-10; Plummer, 2019), runjags (version 2.0.4-4; Denwood, 2016) and coda (version 0.19-3; Plummer et al., 2006), as well as the program JAGS (version 4.2.0; Plummer, 2003). To investigate the influence of sociodemiographic variables (see Table 1) on the change in perceived stress, a Bayesian regression model was calculated using rstanarm. For the comparison of the descriptive variables age, gender and relationship status, Bayesian analogs to *t*-tests were calculated. For all other analyses, ANOVA-like models were used. To analyze changes from T_0_ to T_1_ dependent on mental disorder, difference scores were calculated (T_1_ minus T_0_, see Kruschke, 2018) and disorder group was employed as between factor. To estimate population parameters of interest, Bayesian hierarchical data analyses and parameter estimation were applied. We used adapted and modified scripts from Kruschke (2018). For all analyses, robust hierarchical models were chosen with non-committal priors, allowing the estimation of a wide range of population parameters. Specifically, noise distributions of dependent variables were described by the flexible t-distribution, allowing for outliers through the estimation of the normality parameter ν, which was estimated with an exponential distribution with λ = 1/30. In the case of single group analyses, possible parameters for μ were estimated with a normal distribution and possible parameters for σ with a uniform distribution. For multigroup analyses, individual σ parameters for each group were calculated using a gamma distribution. For the estimation of deflection parameters β (i.e., regression coefficients for each group), normal distributions were employed. For more details, see Kruschke (2014).

**TABLE 1:** Descriptive statistics regarding gender, age, relationship status, educational level, the possession of a garden and/or balcony and Covid-19-related behaviors and infections for the total sample and the subsamples.

	**Total sample**	**GAD**	**PA**	**IA**	**SAD**	**DP**	**OCD**	**BDD**	**ED**	**SP**	**other**	**HC**
	***n* = 2233**	***n* = 135**	***n* = 83**	***n* = 30**	***n* = 86**	***n* = 586**	***n* = 47**	***n* = 16**	***n* = 62**	***n* = 6**	***n* = 156**	***n* = 1026**

**Gender**												
Female	1803	117	75	23	63	470	38	14	59	3	132	809
Male	407	17	7	7	20	105	7	2	2	3	23	214
Non-binary	23	1	1	0	3	11	2	0	1	0	1	3
**Age (*SD*)**	33.21 (12.74)	34.47 (13.25)	33.70 (13.63)	37.50 (14.97)	33.41 (11.45)	34.06 (13.45)	28.28 (9.08)	27.50 (9.27)	27.47 (9.60)	33.67 (9.48)	37.79 (13.31)	32.33 (12.19)
*Min*	18	18	18	19	19	18	19	19	18	19	19	18
*Max*	83	69	74	78	56	69	54	49	64	44	76	83
**in a relationship**	1419	88	54	23	55	312	24	9	32	3	106	713
**Educational level**												
No educational attainment	3	0	0	0	0	0	0	0	1	0	1	1
Secondary school certificate	43	4	3	1	4	15	1	0	1	0	3	11
General secondary school certificate	233	21	11	3	10	85	5	4	7	1	15	71
Advanced technical college certificate/general qualification for university	864	55	39	10	35	271	24	8	24	3	38	357
University degree	1068	53	30	16	36	212	16	4	28	2	97	574
Other	22	2	0	0	1	3	1	0	1	0	2	12
**Garden/balcony**												
Garden	638	38	25	12	20	168	9	5	17	0	45	299
Balcony	728	49	26	9	30	196	18	4	24	2	49	321
Garden and balcony	514	30	19	6	13	106	10	4	12	1	46	267
No garden and balcony	353	18	13	3	23	116	10	3	9	3	16	139
**Current infection**	14	0	2	1	0	2	1	1	0	0	2	5
**Past infection**	19	1	0	0	1	3	1	1	0	0	0	12
**Staying in**	1838	118	73	26	72	485	36	11	50	5	124	838
**Time obtaining information (*SD*)**	52.06 (63.86)	62.00 (64.38)	54.67 (58.59)	55.30 (50.34)	61.81 (93.87)	55.19 (78.03)	53.64 (75.68)	42.63 (30.93)	45.26 (41.11)	116.67 (127.38)	49.71 (53.51)	48.31 (53.30)
*Min*	0	1	0	5	0	0	0	5	0	20	0	0
*Max*	720	360	300	240	660	720	301	120	200	360	300	500

*GAD, generalized anxiety disorder; PA, panic disorder and agoraphobia; IA, illness anxiety; SAD, social anxiety disorder; DP, depression; OCD, obsessive–compulsive disorder; ED, eating disorders; BDD, body dysmorphic disorder; SP, schizophrenia spectrum and other psychotic disorders; other, other non-specified mental disorder; HC, healthy controls; *N/n*, sample size; age, age in years; current infection, number of persons currently infected; past infection, number of persons previously infected; staying in, number of persons staying in most of the time due to Covid-19; time obtaining information, time spending on obtaining information about Covid-19 in minutes per day; *SD*, standard deviations; *min*, minimum; *max*, maximum.*

To assess convergence and representativeness of the Markov Chain Monte Carlo results, autocorrelations, Gelman–Rubin statistics (Gelman and Rubin, 1992), trace plots of parameter values of all iterations, overlap of density plots of parameter estimations from each chain, and effective sample size (ESS) were inspected. ESS of 10,000 could be reached for parameters of interest in nearly all analyses. Inspection of the analyses for the variables *PHQ – panic* and *hand disinfection* indicated non-convergence. Both models showed overcompensation for outliers with values for ν close to zero, resulting in also near zero estimations for μ and σ. To prevent overcompensation, this parameter was set to ν ≥ 1 for *PHQ – panic* and ν ≥ 2 for *hand disinfection*.

For the description and inference of the results, the median of the posterior distributions for the estimated population parameters of interest μ, σ, and the effect size δ are reported. Effect size was calculated as δ = μ−0/σ in the case of a single group and as δ = $\mu_{2}-\mu_{1}/\sqrt{\left(\sigma_{1}^{2}+\sigma_{2}^{2}\right)/2}$ in the case of two groups. To estimate the uncertainty of parameters, the 95% most credible values were reported, i.e., the highest density intervals (*HDI*). For hypothesis-testing, a region of practical equivalence (*ROPE*; Kruschke, 2014, 2018) was defined. Due to the novelty of the situation of the Covid-19 pandemic, practical equivalence on raw scores was difficult to define for each variable. Therefore, we decided to define *ROPEs* in terms of effect sizes δ. Since even small effects could indicate clinically meaningful changes and differences, *ROPEs* were set to −0.2 < δ < 0.2, but to avoid false alarms for negligible effect, this is larger than the “half the size of a small effect” rule of thumb (Kruschke, 2014). The null hypothesis can be accepted if the *HDI* values lie completely within the *ROPE* and the alternative can be accepted if *HDI* lies completely outside the *ROPE*. In the case of overlap of *HDI* and *ROPE*, no clear decision for one or the other hypothesis can be made (Kruschke, 2018). To further interpret the data for changes from T_0_ to T_1_, a 0 change was set as a comparison value and the percentage of the posterior distribution of δ delta above / below 0 was estimated. Thus, also trends for effects can be observed and described if large amounts of the probability mass lie on one or the other side of the comparison value, which is especially helpful in the case of inconclusive results. Similarly, percentage of probability mass below, within, and above the *ROPE* will be reported and interpreted. Concerning the group of PA (*n* = 83), *n* = 44 participants reported a panic attack during the last 4 weeks at T_0_ and T_1_ and rated the occurrence of symptoms during this attack. Therefore, only these participants were included in the analyses regarding changes in the level of perceived symptoms during a panic attack.

## Results

### Sample Characteristics

Descriptive statistics are displayed in Table 1. Of the total sample, 80.84% of participants were female. Approximately half of the participants reported a university degree as their highest educational level (47.83%), followed by an advanced technical college certificate or general qualification for university (38.69%), a general secondary school certificate (10.43%), a secondary school certificate (1.93%), other educational level (0.99%) and no educational attainment (0.13%). Nearly two thirds of the participants were in a relationship (63.55%). 28.57% of the participants reported having access to a garden, 32.60% to a balcony and 23.02% to a garden and balcony, while 15.81% reported neither a garden nor a balcony. The mean age of the total sample was 33.21 years, with a range from 18 to 83 years. Of the total sample, *n* = 14 persons were currently infected with Covid-19, while *n* = 19 participants reported a past infection. Approximately 63% of the participants with a mental disorder stated a currently increased need for therapeutic support and about 49% stated slightly to considerably worsened mental health, as compared to November 2019 (see Table 2).

**TABLE 2:** Absolute and relative frequencies of the perceived changes in mental health and the increased need for therapeutic support.

		**Perceived changes in mental health**										**Increased need for therapeutic support?**			
		**Considerably improved**		**Slightly improved**		**Same**		**Slightly worsened**		**Considerably worsened**		**Yes**		**No**	
	***n***	***f***	***%***	***f***	***%***	***f***	***%***	***f***	***%***	***f***	***%***	***f***	***%***	***f***	***%***
Total sample	1207	92	7.62	212	17.56	304	25.19	407	33.72	192	15.91	444	36.80	763	63.20
GAD	135	8	5.93	26	19.26	24	17.78	54	40.00	23	17.04	61	45.20	74	54.80
PA	83	5	6.02	18	21.69	15	18.07	25	30.12	20	24.10	30	36.10	53	63.90
IA	30	2	6.67	1	3.33	6	20.00	15	50.00	6	20.00	12	40.00	18	60.00
SAD	86	7	8.14	17	19.77	26	30.23	29	33.72	7	8.14	24	27.90	62	72.10
DP	586	48	8.19	113	19.28	88	15.02	218	37.20	119	20.31	269	45.90	317	54.10
OCD	47	5	10.64	10	21.28	15	31.91	13	27.66	4	8.51	16	34.00	31	66.00
BDD	16	0	0	3	18.75	6	37.50	6	37.50	1	6.25	4	25.00	12	75.00
ED	62	4	6.45	13	20.97	6	9.68	28	45.16	11	17.74	23	37.10	39	62.90
SP	6	0	0	1	16.67	4	66.67	1	16.67	0	0	1	16.70	5	83.30
other	156	13	8.33	10	6.41	114	73.08	18	11.54	1	0.64	4	2.60	152	97.40

*n, sample size; f, absolute frequencies; %, relative frequencies; GAD, generalized anxiety disorder; PA, panic disorder and agoraphobia; IA, illness anxiety; SAD, social anxiety disorder; DP, depression; OCD, obsessive–compulsive disorder; BDD, body dysmorphic disorder; ED, eating disorders; SP, schizophrenia spectrum and other psychotic disorders; other, other non-specified mental disorder; HC, healthy controls.*

A Bayesian analog to *t*-test of all participants with self-identified mental disorders (MD) and HC indicated that the HC group was older, with a small effect size (μ_*HC*_ = 31.192; μ_*MD*_ = 33.017; δ = 0.157). Despite the small difference in age between MD and HC, neither age nor any of the other sociodemographic variables showed any effect on the perceived changes in stress, with the exception of relationship status: A Bayesian multiple regression revealed a small effect for relationship status with a median for *b* = −0.288, 95% HDI [−0.581, −0.016] and 97.7% of the posterior below 0, indicating lower perceived stress in participants who were not in a relationship.

### Differences Between T_0_ and T_1_ Regarding Symptom Severity

From all questionnaires, the alternative hypothesis was only met for the DASS-D, showing that DP participants reported a greater number of symptoms, and more severe symptoms, at T_1_ than at T_0_. All other questionnaires yielded inconclusive findings, as the *HDI* partially overlapped with the *ROPE*. However, the results from the PSWQ, WI and FKS indicated a trend toward a higher level of perceived symptoms at T_1_ compared to T_0_, revealing that more than 95% of the posterior distribution was above zero and more than 74% of the posterior distribution was higher than the upper limit of the *ROPE*. For the FKS, the median estimation of δ indicated a medium effect size of 0.61. However, the range of the *HDI* was quite large, which may have been caused by the uncertainty due to the small sample size of BDD.

The remaining questionnaires showed a tendency toward no substantial changes between T_0_ and T_1_, as a high percentage of the posterior distributions lay within the *HDI*. The summary for Bayesian posterior distributions regarding changes in disorder-specific questionnaires is presented in Table 3.

**TABLE 3:** Summary statistics for Bayesian posterior distributions regarding changes in disorder-specific questionnaires (T_1_ minus T_0_).

			***HDI*_μ_**			***HDI*_δ_**			**% of δ**		
**Questionnaire**	**μ**	**σ**	***LL***	***UL***	**δ**	***LL***	***UL***	**% of δ > 0**	**< ROPE**	**in ROPE**	**> ROPE**
**PSWQ**	1.97	7.64	0.59	3.30	0.26	0.09	0.44	99.76	0	25.73	74.27
**PHQ - panic**	0.15	0.95	−0.18	0.61	0.16	−0.19	0.52	81.51	2.36	56.19	41.45
**WI**	0.75	2.25	−0.13	1.69	0.34	−0.06	0.73	95.05	0	23.29	76.22
**SIAS**	0.52	6.54	−1.07	2.02	0.08	−0.16	0.32	74.94	2.57	82.96	16.11
**SPS**	0.74	4.50	−0.44	1.88	0.17	−0.10	0.43	89.23	0.21	59.81	39.96
**DASS-D**	2.34	5.87	1.85	2.81	0.40	0.31	0.48	100	0	0	100
**Y-BOCS**											
Total score	0.31	4.22	−1.10	1.64	0.08	−0.26	0.44	67.33	3.87	71.03	24.18
Obsessions	0.22	2.83	−0.67	1.08	0.08	−0.22	0.41	69.29	0.42	73.71	22.46
Compulsions	−0.08	2.35	−0.82	0.66	−0.03	−0.35	0.28	41.56	0.74	77.94	7.11
**FKS**	2.89	4.85	0.26	5.62	0.61	0.05	1.20	98.31	0.32	7.90	91.88
**EDE-Q**											
Total score	0.08	1.03	−0.18	0.37	0.08	−0.18	0.35	72.80	0	78.99	18.93
Restraint	0.04	1.46	−0.36	0.42	0.03	−0.24	0.30	58.26	0	84.63	10.69
Eating concerns	0.12	1.18	−0.20	0.43	0.11	−0.16	0.37	78.75	0	75.23	23.66
Weight concerns	0.08	1.10	−0.22	0.37	0.07	−0.19	0.35	69.97	0	80.72	16.97
Shape concerns	0.15	0.91	−0.09	0.39	0.17	−0.10	0.43	88.98	0	59.06	40.56
**CAHSA**	0.06	0.95	−0.99	1.03	0.06	−0.81	0.90	55.75	2.20	35.92	37.43

*μ, median of posterior distribution of μ; σ, median of posterior distribution of σ; δ, median of posterior distribution of δ; *HDI*_μ_, 95% Highest Density Interval for μ with LL, lower limit and UL, upper limit; *HDI*_δ_, 95% Highest Density Interval for δ with LL, lower limit and UL, upper limit; % of δ > 0, percentage of the posterior distribution that is greater than the comparison value 0, i.e., no increase/decrease; % of δ < ROPE, percentage of the posterior distribution less than the lower limit; % of δ in ROPE, percentage of the posterior distribution in the interval; % of δ > ROPE, percentage of the posterior distribution higher than the upper limit. PSWQ, Penn-State Worry Questionnaire; PHQ - panic, Patient Health Questionnaire – panic subscale; WI, Whiteley Index; SPS, Social Phobia Scale; SIAS, Social Interaction Anxiety Scale; DASS-D, Depression Anxiety Stress Scale – Depression Subscale; Y-BOCS, Yale-Brown Obsessive Compulsive Scale; FKS, Body Dysmorphic Symptoms Inventory; EDE-Q, Eating Disorder Examination Questionnaire; CAHSA, Continuum of Auditory Hallucinations – State Assessment.*

### Differences Between T_0_ and T_1_ Regarding Perceived Stress

Table 4 depicts the results of the Bayesian posterior distributions for changes in perceived stress. For the groups of GAD, PA, DP, BDD, ED, and HC, the alternative hypothesis was met, showing higher psychosocial stress symptoms at T_1_ compared to T_0_.

**TABLE 4:** Summary statistics for Bayesian posterior distributions regarding changes on the subscale stress of the PHQ (T_1_ minus T_0_).

			***HDI*_μ_**			***HDI*_δ_**			**% of δ**		
**Groups**	**μ**	**σ**	***LL***	***UL***	**δ**	***LL***	***UL***	**% of δ > 0**	**< ROPE**	**in ROPE**	**> ROPE**
**GAD**	1.58	2.98	1.10	2.06	0.53	0.37	0.72	100	0	0	100
**PA**	1.49	2.68	0.96	2.01	0.55	0.35	0.77	100	0	0.02	99.99
**IA**	1.24	2.69	0.54	1.96	0.46	0.19	0.75	99.90	0	3.19	96.81
**SAD**	1.03	2.73	0.45	1.54	0.38	0.17	0.57	99.98	0	4.96	95.05
**DP**	1.55	3.10	1.27	1.81	0.50	0.41	0.59	100	0	0	100
**OCD**	0.92	2.79	0.16	1.56	0.33	0.06	0.57	98.64	0	17.21	82.74
**BDD**	1.64	2.62	0.88	2.60	0.63	0.30	1.06	100	0	0.30	99.71
**ED**	1.38	2.75	0.80	1.97	0.50	0.28	0.74	100	0	0.33	99.68
**SP**	1.35	2.62	0.45	2.30	0.52	0.14	0.94	99.52	0	3.77	96.18
**Other**	0.83	2.43	0.41	1.24	0.34	0.17	0.52	100	0	5.55	94.46
**HC**	1.56	2.66	1.37	1.73	0.58	0.51	0.65	100	0	0	100
**Contrast MD vs. HC**	−0.25		−0.55	0.02	−0.09	−0.20	0.01	4.03	2.44	97.57	0

*μ, median of posterior distribution of μ; σ, median of posterior distribution of σ; δ, median of posterior distribution of δ; *HDI*_μ_, 95% Highest Density Interval for μ with LL, lower limit and UL, upper limit; *HDI*_δ_, 95% Highest Density Interval for δ with LL, lower limit and UL, upper limit; % of δ > 0, percentage of the posterior distribution that is greater than the comparison value 0, i.e., no increase/decrease; % of δ < ROPE, percentage of the posterior distribution less than the lower limit; % of δ in ROPE, percentage of the posterior distribution in the interval; % of δ > ROPE, percentage of the posterior distribution higher than the upper limit. GAD, generalized anxiety disorder; PA, panic disorder and agoraphobia; IA, illness anxiety; SAD, social anxiety disorder; DP, depression; OCD, obsessive–compulsive disorder; BDD, body dysmorphic disorder ED, eating disorders; SP, schizophrenia spectrum and other psychotic disorders; other, other non-specified mental disorder; HC, healthy controls; Contrast MD vs. HC, contrast between all participants with a mental disorder (=MD; includes all groups with mental disorders from GAD to other) and healthy controls.*

With the exception of the groups of IA, SAD, OCD, and SP, 100% of the posterior distribution was higher than zero for all groups. Furthermore, for the groups of IA, SAD and other, more than 95% of the posterior distribution was higher than the upper limit of the *ROPE*, which also indicates a trend toward an increase on the stress subscale from T_0_ to T_1_. For both OCD and SP, more than 98% of the posterior distribution was higher than zero and about 82% (OCD) and 94% (SP) of the posterior distribution lay above the upper limit of the *ROPE*, respectively. This indicates a tendency toward higher perceived stress levels at T_1_ compared to T_0_.

For the contrast of MD and HC, the null hypothesis could be accepted, as *HDI* lay completely within the *ROPE* and with approximately 97% of the posterior distribution of δ.

### Differences Between T_0_ and T_1_ Regarding Behaviors Related to Covid-19

Table 5 displays the Bayesian posterior distributions regarding behaviors related to hygiene, social contacts and grocery shopping. Concerning the amount of change in hand washing, the alternative hypothesis was met for all groups, with all participants reporting a higher frequency of hand washing at T_1_ than at T_0_. Contrast analysis revealed that MD did not clearly differ from HC in terms of the frequency of hand washing. Furthermore, the two groups did not differ regarding the amount of change in hand disinfection. While GAD, PA, SAD, DP, ED, other and HC showed an increased hand disinfection from T_0_ to T_1_, IA, OCD, and SP revealed a trend toward a change in the frequency of hand disinfection.

**TABLE 5:** Summary statistics for Bayesian posterior distributions regarding changes in behaviors related to hygiene, contacts and grocery shopping (T_1_ minus T_0_).

			***HDI*_μ_**			***HDI*_δ_**			**% of δ**		
	**μ**	**σ**	***LL***	***UL***	**δ**	***LL***	***UL***	**% of δ > 0**	**< ROPE**	**in ROPE**	**> ROPE**
**Hand washing**											
GAD	2.64	2.37	2.32	3.08	1.12	0.89	1.37	100	0	0	100
PA	2.62	2.28	2.26	3.10	1.16	0.89	1.45	100	0	0	100
IA	2.55	2.39	2.08	3.08	1.06	0.73	1.42	100	0	0	100
SAD	2.60	2.56	2.22	3.12	1.02	0.78	1.30	100	0	0	100
DP	2.42	2.12	2.19	2.62	1.14	1.01	1.27	100	0	0	100
OCD	2.61	2.73	2.19	3.22	0.96	0.64	1.30	100	0	0	100
BDD	2.61	2.16	2.16	3.21	1.21	0.83	1.77	100	0	0	100
ED	2.55	2.07	2.16	2.98	1.24	0.94	1.59	100	0	0	100
SP	2.53	2.18	2.01	3.13	1.16	0.71	1.71	100	0	0	100
other	2.67	2.15	2.36	3.07	1.25	1.01	1.52	100	0	0	100
HC	2.45	2.04	2.28	2.60	1.20	1.09	1.30	100	0	0	100
Contrast MD vs. HC	0.14		−0.08	0.42	0.06	−0.03	0.20	87.91	0	97.18	2.82
**Time washing hands**											
GAD	3.30	3.67	2.55	4.16	0.90	0.66	1.20	100	0	0	100
PA	3.19	3.67	2.40	4.18	0.87	0.58	1.20	100	0	0	100
IA	2.61	3.28	1.53	3.47	0.78	0.41	1.17	100	0	0.09	99.92
SAD	2.97	2.77	2.35	3.72	1.08	0.75	1.45	100	0	0	100
DP	2.72	2.72	2.42	3.01	1.00	0.87	1.14	100	0	0	100
OCD	2.83	4.26	1.98	3.87	0.67	0.38	1.00	100	0	0	99.98
BDD	2.64	2.36	1.76	3.45	1.12	0.53	1.97	100	0	0	100
ED	2.62	2.29	2.01	3.23	1.14	0.78	1.60	100	0	0	100
SP	2.86	3.03	1.84	4.20	0.95	0.40	1.66	100	0	0	99.94
Other	2.80	2.80	2.28	3.37	1.00	0.77	1.25	100	0	0	100
HC	2.50	2.33	2.28	2.71	1.07	0.96	1.18	100	0	0	100
Contrast MD vs. HC	0.37		−0.02	0.77	0.13	−0.01	0.28	97.57	0	81.34	18.66
**Hand disinfection**											
GAD	0.69	1.65	0.40	1.04	0.42	0.22	0.64	100	0	0.47	99.53
PA	0.57	1.43	0.33	0.88	0.40	0.21	0.62	100	0	0.98	99.02
IA	0.45	1.34	0.12	0.78	0.34	0.09	0.61	99.33	0.02	11.90	88.09
SAD	0.43	1.03	0.21	0.64	0.42	0.21	0.62	99.99	0	2.00	98.00
DP	0.41	1.06	0.31	0.51	0.39	0.29	0.48	100	0	0.03	99.98
OCD	0.44	1.25	0.16	0.74	0.35	0.12	0.59	99.77	0	8.26	91.75
BDD	0.52	1.56	0.19	0.98	0.33	0.11	0.67	99.69	0.02	11.12	88.87
ED	0.46	1.12	0.23	0.72	0.41	0.20	0.65	99.98	0	2.41	97.59
SP	0.49	1.26	0.14	0.92	0.39	0.07	0.80	99.36	0.09	8.25	91.67
Other	0.55	1.42	0.35	0.79	0.38	0.24	0.56	100	0	0.46	99.55
HC	0.40	1.00	0.33	0.48	0.40	0.33	0.47	100	0	0	100
Contrast MD vs. HC	0.10		−0.03	0.25	0.09	-0.02	0.22	93.04	0	94.82	5.18
**Social contacts**											
GAD	−9.47	7.58	−11.27	−7.77	−1.25	−1.56	−0.95	0	100	0	0
PA	−9.50	8.86	−12.26	−6.70	−1.07	−1.41	−0.74	0	100	0	0
IA	−10.48	7.87	−13.45	−7.39	−1.34	−1.97	−0.82	0	100	0	0
SAD	−9.57	7.85	−11.79	−7.29	−1.22	−1.62	−0.89	0	100	0	0
DP	−10.26	7.77	−11.18	−9.40	−1.32	−1.48	−1.16	0	100	0	0
OCD	−8.13	7.84	−11.03	−5.34	−1.04	−1.49	−0.65	0	100	0	0
BDD	−13.17	9.04	−18.89	−8.15	−1.45	−2.29	−0.76	0	100	0	0
ED	−11.55	8.11	−14.20	−9.04	−1.43	−1.92	−1.02	0	100	0	0
SP	−12.60	9.25	−21.38	−6.45	−1.35	−2.41	−0.56	0	99.91	0.09	0
other	−13.58	10.06	−15.85	−11.39	−1.35	−1.66	−1.05	0	100	0	0
HC	−15.00	9.75	−15.88	−14.10	−1.54	−1.68	−1.40	0	100	0	0
Contrast MD vs. HC	4.11		2.62	5.60	0.45	0.28	0.61	100	0	0.40	99.61
**Grocery shopping**											
GAD	−1.36	1.31	−1.62	−1.12	−1.04	−1.30	−0.81	0	100	0	0
PA	−1.30	1.23	−1.57	−1.07	−1.06	−1.34	−0.82	0	100	0	0
IA	−1.28	1.21	−1.66	−1.01	−1.06	−1.45	−0.78	0	100	0	0
SAD	−1.27	1.21	−1.53	−1.06	−1.05	−1.31	−0.83	0	100	0	0
DP	−1.15	1.24	−1.26	−1.04	−0.92	−1.04	−0.82	0	100	0	0
OCD	−1.09	1.29	−1.35	−0.73	−0.83	−1.08	−0.55	0	100	0	0
BDD	−1.26	1.25	−1.69	−0.95	−1.01	−1.42	−0.70	0	100	0	0
ED	−1.16	1.33	−1.41	−0.85	−0.86	−1.10	−0.61	0	100	0	0
SP	−1.23	1.23	−1.69	−0.88	−1.00	−1.45	−0.65	0	99.98	0	0
other	−1.13	1.30	−1.31	−0.91	−0.87	−1.05	−0.68	0	100	0	0
HC	−1.15	1.10	−1.23	−1.07	−1.05	−1.14	−0.96	0	100	0	0
Contrast MD vs. HC	−0.07		−0.21	0.05	−0.06	−0.18	0.04	12.98	1.75	98.25	0

*μ, median of posterior distribution of μ; σ, median of posterior distribution of σ; δ, median of posterior distribution of δ; *HDI*_μ_, 95% Highest Density Interval for μ with LL, lower limit and UL, upper limit; *HDI*_δ_, 95% Highest Density Interval for δ with LL, lower limit and UL, upper limit; % of δ > 0, percentage of the posterior distribution that is greater than the comparison value 0, i.e., no increase/decrease; % of δ < ROPE, percentage of the posterior distribution less than the lower limit; % of δ in ROPE, percentage of the posterior distribution in the interval; % of δ > ROPE, percentage of the posterior distribution higher than the upper limit; hand washing, frequency of hand washing per day; time hand washing, time spent washing hands in minutes per day; hand disinfection, frequency of hand disinfection per day; social contacts, number of social contacts in real life per week; grocery shopping, frequency of grocery shopping per week; GAD, generalized anxiety disorder; PA, panic disorder and agoraphobia; IA, illness anxiety; SAD, social anxiety disorder; DP, depression; OCD, obsessive–compulsive disorder; BDD, body dysmorphic disorder ED, eating disorders; SP, schizophrenia spectrum and other psychotic disorders; other, other non-specified mental disorder; HC, healthy controls; Contrast MD vs. HC, contrast between all participants with a mental disorder (=MD; includes all groups with mental disorders from GAD to other) and healthy controls.*

Regarding the time spent on hand washing, all alternative hypotheses were accepted, and more than 99.90% of the posterior distribution was higher than the upper limit of the *ROPE* for all groups. This indicates an increased number of minutes spent on hand washing per day at T_1_ compared to T_0_. However, this increased amount of time did not appear to differ between MD and HC from T_0_ to T_1_, as approximately 81% of the posterior distribution was within the *ROPE*, although the *HDI* was not entirely enclosed by the *ROPE*.

All groups revealed having fewer social contacts at T_1_ compared to T_0_. Moreover, the alternative hypothesis for the contrast MD vs. HC was accepted. Approximately 99% of the posterior distribution lay above the upper limit of the *ROPE*, indicating that HC reported a stronger decrease in social contacts than did MD.

For grocery shopping, the analysis revealed that all groups showed a decreased frequency of grocery shopping per week at T_1_ compared to T_0_ (all *HDI*s completely outside the *ROPE*). The contrast between MD and HC indicated no substantial differences, as the *HDI* was completely inside the *ROPE*.

### Perceived Worries, Fears and Quality of Life

The summary for Bayesian posterior distributions and contrast analyses regarding personal and general worries, perceived risk and fear of an infection as well as changes in quality of life related to Covid-19 are presented in Tables 6, 7.

**TABLE 6:** Summary statistics for Bayesian posterior distributions regarding personal and general worries, perceived risk and fear of an infection and quality of life related to Covid-19.

			***HDI*_μ_**	
	**μ**	**σ**	***LL***	***UL***
**Personal worries**				
GAD	3.52	1.10	3.34	3.71
PA	3.14	1.16	2.90	3.37
IA	3.37	1.08	3.02	3.72
SAD	3.00	1.14	2.77	3.23
DP	3.15	1.10	3.06	3.24
OCD	3.07	1.15	2.78	3.37
BDD	3.11	1.07	2.70	3.51
ED	3.14	0.99	2.91	3.37
SP	3.01	1.11	2.46	3.53
Other	2.68	1.04	2.52	2.85
HC	2.78	0.97	2.72	2.84
**General worries**				
GAD	3.86	0.85	3.71	4.01
PA	3.73	0.93	3.58	3.91
IA	3.70	0.90	3.48	3.91
SAD	3.67	0.96	3.49	3.82
DP	3.75	0.97	3.67	3.82
OCD	3.57	0.98	3.29	3.77
BDD	3.69	0.89	3.44	3.92
ED	3.81	0.89	3.64	4.00
SP	3.69	0.91	3.40	3.96
Other	3.67	0.92	3.54	3.79
HC	3.68	0.85	3.63	3.74
**Risk of infection**				
GAD	3.04	0.88	2.94	3.12
PA	3.06	0.91	2.97	3.19
IA	3.06	0.90	2.95	3.21
SAD	3.03	0.89	2.90	3.12
DP	3.05	0.93	3.00	3.12
OCD	3.04	0.88	2.89	3.13
BDD	3.05	0.91	2.93	3.21
ED	3.07	0.92	2.97	3.23
SP	3.05	0.91	2.93	3.22
Other	3.05	0.92	2.96	3.15
HC	3.04	0.92	2.99	3.09
**Fear of infection**				
GAD	3.04	1.14	2.85	3.23
PA	2.83	1.21	2.57	3.07
IA	3.49	1.16	3.05	3.92
SAD	2.60	1.07	2.38	2.83
DP	2.36	1.07	2.28	2.45
OCD	2.68	1.18	2.36	3.01
BDD	2.53	1.16	2.03	3.03
ED	2.36	1.17	2.08	2.66
SP	2.56	1.10	1.88	3.17
Other	2.31	1.00	2.16	2.47
HC	2.16	0.94	2.10	2.22
**Quality of life**				
GAD	3.74	1.09	3.57	3.92
PA	3.60	1.21	3.39	3.82
IA	3.63	1.18	3.33	3.93
SAD	3.34	1.11	3.11	3.56
DP	3.63	1.21	3.53	3.72
OCD	3.50	1.18	3.23	3.76
BDD	3.62	1.14	3.27	3.97
ED	3.49	1.23	3.23	3.73
SP	3.49	1.16	3.04	3.88
Other	3.42	1.16	3.25	3.59
HC	3.74	0.96	3.69	3.80

*μ, median of posterior distribution of μ; σ, median of posterior distribution of σ; δ, median of posterior distribution of δ; *HDI*_μ_, 95% Highest Density Interval for μ with LL, lower limit and UL, upper limit; personal worries, rating of the perceived worries about personal consequences due to Covid-19; general worries, rating of the perceived worries about consequences for society due to Covid-19; risk of infection, rating of the perceived risk of contracting Covid-19; fear of infection, rating of the perceived fear of contracting Covid-19; GAD, generalized anxiety disorder; PA, panic disorder and agoraphobia; IA, illness anxiety; SAD, social anxiety disorder; DP, depression; OCD, obsessive–compulsive disorder; BDD, body dysmorphic disorder; ED, eating disorders; SP, schizophrenia spectrum and other psychotic disorders; other, other non-specified mental disorder; HC, healthy controls.*

**TABLE 7:** Summary statistics for Bayesian posterior distributions for contrast between participants with and without mental disorders regarding personal and general worries, perceived risk and fear of an infection and quality of life related to Covid-19.

		***HDI*_μ_**			***HDI*_δ_**			**% of δ**		
	**μ**	***LL***	***UL***	**δ**	***LL***	***UL***	**% of δ > 0**	**< ROPE**	**in ROPE**	**> ROPE**
**Personal worries**										
Contrast MD vs. HC	0.33	0.22	0.46	0.32	0.21	0.44	100	0	1.78	98.22
**General worries**										
Contrast MD vs. HC	0.03	−0.06	0.11	0.03	−0.06	0.13	73.34	0	99.98	0.02
**Risk of infection**										
Contrast MD vs. HC	0.01	−0.05	0.08	0.01	−0.06	0.08	61.33	0	100	0
**Fear of infection**										
Contrast MD vs. HC	0.52	0.39	0.65	0.50	0.37	0.63	100	0	0	100
**Life quality**										
Contrast MD vs. HC	−0.20	−0.31	−0.08	−0.19	−0.29	−0.08	0.03	39.49	60.51	0

*μ, median of posterior distribution of μ; σ, median of posterior distribution of σ; δ, median of posterior distribution of δ; *HDI*_μ_, 95% Highest Density Interval for μ with LL, lower limit and UL, upper limit; *HDI*_δ_, 95% Highest Density Interval for δ with LL, lower limit and UL, upper limit; % of δ > 0, percentage of the posterior distribution that is greater than the comparison value 0, i.e., no increase/decrease; % of δ < ROPE, percentage of the posterior distribution less than the lower limit; % of δ in ROPE, percentage of the posterior distribution in the interval; % of δ > ROPE, percentage of the posterior distribution higher than the upper limit; personal worries, rating of the perceived worries about personal consequences due to Covid-19; general worries, rating of the perceived worries about consequences for society due to Covid-19; risk of infection, rating of the perceived risk of contracting Covid-19; fear of infection, rating of the perceived fear of contracting Covid-19; Contrast MD vs. HC, contrast between all participants with a mental disorder (=MD; includes all groups with mental disorders from GAD to other) and healthy controls.*

Null hypotheses for the differences between MD and HC regarding perceived general worries and the perceived risk of an infection were accepted, as the *HDIs* were completely inside the *ROPEs*.

Concerning the fear of infection with Covid-19 and the perceived worries about personal consequences due to Covid-19, alternative hypotheses were accepted. More specifically, MD revealed higher fear of an infection and worries about the personal consequences of Covid-19 than did HC. Regarding quality of life, the results were inconclusive. No clear trend toward a difference between MD and HC was found.

## Discussion

The present study examined potential changes in symptom severity, perceived stress levels, behaviors related to Covid-19, worries, fears and quality of life in individuals with and without mental disorders in an exploratory manner during the initial outbreak of Covid-19.

First, regarding symptom severity, an increase in symptom severity was found for the group of DP, BDD, IA, and GAD during the outbreak of Covid-19 compared to November 2019. For the group of DP, this result may be corroborated by theoretical models on the etiology and maintenance of depression, such as the relevance of critical und uncontrollable events in terms of learned helplessness (Abramson et al., 1978) and the lack of response-contingent positive reinforcement (Lewinsohn, 1974). In our study, participants with DP may have experienced a loss of positive reinforcement, which could be attributed to the fact that nearly 82% reported staying at home most of the time during the outbreak of Covid-19. This behavior may have led to a further loss of positive reinforcement and enhanced depressive symptoms, because the imposed restrictions to reduce the risk of a Covid-19-infection included prohibitions on social activities, such as seeing friends or visiting sports clubs. However, the interpretation of the present results is limited by the fact that our study assessed depressive symptoms in the group of DP only. Thus, it remains unclear whether the other mental disorders and HC may also have reported an increase in depressive symptoms due to Covid-19.

For BDD, the results indicated a trend toward an increase in symptom severity between November 2019 and the time of survey completion. In line with cognitive-behavioral models for BDD (e.g., Wilhelm et al., 2013), spending time at home in isolation may have led to even stronger selective attention toward problematic body parts. In turn, this may have activated an increase in emotions such as shame and anxiety and BDD symptoms. Moreover, as in BDD, we found a trend toward an increase in symptoms in individuals with self-reported GAD and IA, which may result from the illness-related cognitions of these mental disorders as a core feature (American Psychiatric Association, 2018). During the spread of Covid-19, both groups may have been confronted with their greatest worries (e.g., contracting a disease). This, in turn, may have increased their perceived symptoms. Yet, it is unknown whether the increase in disorder-specific anxiety is specific to these two groups, as other studies observed general anxiety to be a common reaction to pandemics in the general population (e.g., Leung et al., 2005; Li et al., 2020a; Mazza et al., 2020; Wang et al., 2020a).

Perhaps surprisingly, no changes in the level of OCD and ED symptoms were found from November 2019 to the current outbreak of Covid-19. This stands in contrast to recent studies in patients with ED (Castellini et al., 2020; Schlegl et al., 2020) and OCD (Davide et al., 2020) before and during the outbreak of Covid-19, which detected greater impairments in both mental disorders. Although in the present study, about 67% of the posterior distribution for OCD and 72% of the posterior distribution for ED was greater than zero, most of the posteriors lay within the *ROPE*, indicating no trend toward a change in pathology. Moreover, for SP, the level of psychotic symptoms did not differ between the two assessed time points. This is in contrast to a recent review on pandemics (Brown et al., 2020), which assumed an association between a higher risk of onset of a psychotic episode and psychosocial stress due to a pandemic. Presumably, this may be due to group size, as estimations become more precise as group sizes increase. Since the group of SP was very small in the present study, a possible difference may be more pronounced in a larger sample. All of the other analyses on the remaining mental disorders suggested no substantial changes in symptom severity.

The second aim of this study was to examine the perceived stress levels before and during the onset of Covid-19. We observed a substantial effect for the groups of GAD, PA, DP, BDD, ED, and HC, while for all other groups there was a trend toward higher perceived stress levels during the spread of Covid-19. This is in line with studies on earlier pandemics, which found high stress levels in the general population (Lau et al., 2005; Taylor et al., 2008). As no previous studies have examined stress level symptoms in participants with mental disorders before and during a pandemic, the present study is the first to underline the results from the general population in participants with mental disorders. Furthermore, we found no differences between participants with and without mental disorders regarding changes in stress level symptoms between the outbreak of Covid-19 and before the pandemic. This seems to be in contrast to a recent study on anxiety-related and mood disorders, which found higher Covid-19-related stress in the anxiety group than in participants with mood disorders and healthy controls (Asmundson et al., 2020). However, Asmundson et al. (2020) only compared the recent level of stress related to Covid-19, while the present study investigated the level of change in psychosocial stress between November 2019 and the time of the survey. Thus, as found in the present study, the experience of Covid-19 may increase psychosocial stress in people with and without mental disorders to a comparable degree, while the level of stress may differ between mental disorders and HC, as found by Asmundson et al. (2020).

The third aim was to investigate potential changes in behaviors related to Covid-19. Across all groups, participants with and without mental disorders seemed to implement behaviors which were recommended to avoid contracting Covid-19. These results are in line with Lau et al. (2005), who reported preventive behavior in 66.7% of their total sample during the SARS outbreak of 2003 in Hong Kong. In our study, the results indicated a higher frequency of hand washing and more time spent on hand washing for all participants with mental disorders. Moreover, all participants showed a higher increase in their frequency of hand washing between November 2019 and the current situation. The analysis for hand disinfection revealed that GAD, PA, SAD, DP, ED, other and HC showed an increased frequency of hand disinfection during the outbreak of Covid-19 compared to November 2019. No differences were found between participants with mental disorders and HC regarding the frequency of hand washing and disinfection as well as the time spent on hand washing. One may also have expected an increase in the frequency of hand disinfection for the group of IA and OCD, as persons suffering from these disorders fear contracting diseases. For these groups, medians for δ > 0.30 were observed, but the length of *HDI* was substantial. Only a trend in the expected direction was observed. The results for hand washing and disinfection may explain the lack of increase of OCD symptoms within the respective group, which is in contrast to the study by Davide et al. (2020). In our study, a higher level of hand washing and hand disinfection was observed among many groups during the outbreak of Covid-19 compared to November 2019. Washing and disinfection behaviors are related to OCD (American Psychiatric Association, 2018). In the current outbreak of Covid-19, these behaviors may have been considered as a normal reaction, explaining our findings of increased hand washing and disinfection in nearly all groups, but no substantial change in the symptom severity of OCD.

Regarding grocery shopping and social contacts, these behaviors also seemed to be influenced by restrictions due to Covid-19, with all groups reporting a lower frequency of both behaviors during the outbreak of Covid-19 than before. While all participants had fewer contacts during the outbreak of Covid-19, the reduction in the number of contacts was greater for HC than for participants with mental disorders. Moreover, the results indicated that participants with mental disorders worried more about personal consequences due to Covid-19 than did HC and showed a higher fear of infection. However, the outbreak of Covid-19 seems to influence the perceived risk of an infection as well as quality of life equally in participants with mental disorders and HC.

The current study has some limitations. First, sample sizes of IA, BDD and SP were quite small, which may have introduced a greater *HDI* and therefore a greater uncertainty. As reported prevalences for these disorders are quite small (IA: 1.3–10%; BDD: 2.4%; SP: 0.3–0.7%; American Psychiatric Association, 2018), our sample sizes may reflect the proportion in the general population. Sample sizes should be increased for more accurate estimates. Moreover, dropouts on the landing page and during the study were quite high (presumed reasons: curiosity, comparatively long duration, put off by the length of the informed consent). Nonetheless, it is not unusual for online studies to report larger dropouts than lab-based studies (Birnbaum, 2004; Hochheimer et al., 2019).

Second, data for November 2019 were assessed retrospectively, which might have introduced a memory bias in terms of remembering disorder-specific symptoms, stress and behaviors. A study by Safer and Keuler (2002) reported that psychotherapy patients reliably recalled their pre-therapy distress, but also found a bias toward overestimating pre-therapy distress, while healthy individuals showed no over- or underestimation of recalled distress. Other research has reported similar results regarding an overestimation of recalled emotions (e.g., Smith et al., 2006, 2008; Levine et al., 2009). In the current study, a recall of the number of life events was implemented (e.g., looking at one’s calendar, photos on one’s smartphone, and diaries) to help participants to remember their thoughts, feelings and behaviors. Due to the sudden spread of Covid-19, it was not possible to implement a longitudinal design with our participants before the outbreak, and the present approach was the best procedure available to assess potential deterioration in mental disorders and healthy controls. However, further studies are needed concerning the longitudinal impact of pandemics like Covid-19 on the prevalence and course of mental disorders. The findings of this study showed a clear trend toward an association of Covid-19 with a meaningful worsening of symptoms for DP, GAD, IA, and BDD. However, it remains unclear how symptoms of mental disorders behave over the course of time. From a neuroanatomical perspective, studies on the impact of pandemics on genetic liability (e.g., Fusar-Poli et al., 2014) and neuronal networks might be a promising approach, as symptoms may worsen the longer the pandemic continues.

Third, participants were assigned to their groups by self-identifying their mental disorder. A clinical diagnosis or a structured clinical interview would have been the gold standard, and biased self-identification in the present study cannot be completely excluded. In line with the procedure of Hartmann et al. (2019), we provided short descriptions of the respective mental disorders based on the main criteria of the Diagnostic and Statistical Manual of Mental Disorders – fifth edition (American Psychiatric Association, 2018) and asked participants to select their experienced disorder. It cannot be ruled out that participants actually suffered from another mental disorder or chose the mental disorder with the highest current burden in the case of comorbidity. For instance, participants may have selected DP when they were actually suffering from a bipolar disorder and currently experiencing a depressive episode. Moreover, previous and current research on pandemics has shown that during pandemics, negative emotions are reported to a higher degree among the general population (e.g., Leung et al., 2005; Li et al., 2020b; Tian et al., 2020), among infected persons (e.g., Cheng et al., 2004; Jeong et al., 2016), and among populations with mental disorders (e.g., Van Rheenen et al., 2020). Therefore, an influence of the participants’ current mood on their self-identification might have created a bias toward a self-diagnosis. Nevertheless, some studies found self-identification to be quite accurate for depressive and bipolar disorder (Kupfer et al., 2002; Sanchez-Villegas et al., 2008; Stuart et al., 2014). Furthermore, as we examined only a particular selection of mental disorders, participants may have chosen a specific mental disorder as posing the currently highest burden, while actually suffering from a different mental disorder that was not assessed, such as addiction, posttraumatic stress disorder or a personality disorder. In addition, for PA, only symptoms of a panic attack were investigated, while we did not assess avoidance or safety behaviors. Additionally, participants who were partially remitted or subthreshold at the time of the assessment were also included in the respective disorder-specific samples, as it was not possible to determine the time point from which their symptoms had improved. Nevertheless, these participants still reported symptoms belonging to the reported mental disorder, as they were asked to choose the specific mental disorder only if they were currently suffering from it. For future studies, it might be useful to investigate the impact of pandemics on the subgroups or to differentiate between individuals with different medication or treatments related to the specific mental disorders. Furthermore, MD and HC were not matched in this study. Matching groups was not possible because of the short period of time for data collection and conducting the study. Hence, we decided in favor of a large sample. Moreover, more women enrolled in both groups than men, which may reflect a natural gender bias, as women are more likely to be affected by anxiety, eating and mood disorders (American Psychiatric Association, 2018), and are also more likely to participate in studies (Dunn et al., 2004). Furthermore, a higher percentage of HC reported a relationship than MD, which might be explained by the finding that being single is associated with increased rates of different mental disorders in both sexes (Klose and Jacobi, 2004). Finally, the recruitment and assessment method may have led to a selection bias. However, due to the general situation in Germany (e.g., restrictions on social distancing, many inpatient and outpatient treatment facilities were partially or fully closed, people were staying at home), we had to implement an online assessment and could not collect data in the laboratory.

Notwithstanding the aforementioned limitations, the results of the present study give rise to some theoretical and practical implications. In the future, zoonotic pandemics are highly likely, as about 75% of all new emerging diseases are carried from animals to humans (United Nations Environment Program, 2020). Therefore, the influence of pandemics on mental health is set to increase, which emphasizes the need to strengthen current findings and theories. In particular, it might be useful to integrate new findings into a model on the impact of pandemics on mental disorders, which includes risk factors of new incidences and symptom deterioration, disorder-specific features or the impact of social isolation and fears of infection.

Furthermore, as our findings suggest an impact of Covid-19 on people both with and without mental disorders, both groups could benefit from offers of support. For instance, governments could provide the general population with information about typical reactions during a pandemic. This “help for self-help” could include psychoeducation on mood and emotional responses during times of major changes, the impact of isolation, quarantine and loss of positive reinforcement, as well as information on supportive behaviors, such as implementing a daily structure, looking for personal resources at home and retaining social contact via telephone or internet. Moreover, therapy professionals should sensitively consider the impact of a pandemic for each patient individually and try to maintain a therapeutic contact in case of need during a pandemic. As it is often necessary to avoid face-to-face contact, E-Health programs and online therapy seem to be a promising approach, since recent research has found evidence for the efficacy and highlighted the advantages of online therapy or consultation services (e.g., Orman and O’Dea, 2018; Andersson et al., 2019). Other types of online support (e.g., online therapy, online consultation, online self-help groups) should be set up in advance before a pandemic, enabling people to familiarize themselves with them. Furthermore, the implementation of a crisis line for telephone consultation with professionals (e.g., trained persons, psychologists, doctors) for affected people may be useful, especially for people without internet access.

To conclude, the present study was the first to examine the perceived impact of Covid-19 on symptoms of GAD, PA, IA, SAD, DP, OCD, BDD, ED, and SP. It adds knowledge on the perceived impact of pandemics on people with mental disorders. Only DP, GAD, IA, and BDD showed a trend toward an increase in symptom severity as a reaction to Covid-19. All groups revealed higher perceived stress levels during the current situation and reported changes in their behaviors regarding hygiene and a reduced frequency of social contacts and grocery shopping. Additionally, higher personal worries and a higher fear of contracting Covid-19 were found among people with self-identified mental disorders compared to HC. This study emphasizes the need for further studies to investigate the longitudinal impact of Covid-19 on people with and without mental disorders. Future supportive programs could benefit from these results, as they may establish special psychosocial services as a reaction to a pandemic, such as consultation and therapy via internet and telephone.

## Data Availability Statement

The datasets generated for this study are available on request to the corresponding author.

## Ethics Statement

The studies involving human participants were reviewed and approved by Ethics Committee of the Institute of Psychology, University of Münster. Written informed consent for participation was not required for this study in accordance with the national legislation and the institutional requirements.

## Author Contributions

HQ, SV, JS, F-JH, and UB planned and conducted the study. RD and HQ analyzed the data. HQ wrote the first draft of the manuscript. All the authors contributed to the compilation of the manuscript and read and approved the submitted version.

## Conflict of Interest

The authors declare that the research was conducted in the absence of any commercial or financial relationships that could be construed as a potential conflict of interest.
